# Partial arch replacement of type A aortic dissection after thoracic endovascular aortic repair for type B dissection

**DOI:** 10.1186/s43044-023-00412-y

**Published:** 2023-10-06

**Authors:** Tomohiro Nakajima, Yutaka Iba, Keishi Ogura, Nobuyoshi Kawaharada

**Affiliations:** 1 Department of Cardiovascular Surgery, Sapporo Medical University School of Medicine, South-1, West-16, Chuo-ku, Sapporo, 060-8543 Japan; 2 Division of Radiology and Nuclear Medicine, Sapporo Medical University School of Medicine, Sapporo, Japan

**Keywords:** Retrograde type A aortic dissection, Thoracic endovascular aortic repair, Type B aortic dissection

## Abstract

**Background:**

Stent graft-induced new entry (SINE), defined as the stent graft-induced formation of a new entry point for blood to enter an area, is increasingly being observed after thoracic endovascular aortic repair (TEVAR) for Stanford type B aortic dissection worldwide. We herein describe a case of Stanford type A aortic dissection due to proximal SINE after TEVAR for Stanford type B dissection.

**Case presentation:**

This case involved a 58-year-old man with type A aortic dissection due to SINE. Six years previously, he had developed severe back pain and was diagnosed with type B aortic dissection after computed tomography examination. Because the primary entry was positioned at the descending aorta, we conducted TEVAR for exclusion of the entry with a GORE TAG conformable thoracic aortic graft. He was thereafter followed by our hospital. Six years later, he developed jaw pain and was examined at another hospital. He was transferred to our hospital because of the possibility of type A dissection. Computed tomography revealed type A aortic dissection with proximal site SINE. Emergency partial arch replacement was conducted, and he was discharged on postoperative day 27. Because the entry was at the lesser curve of the arch, we excluded the entry and conducted partial arch replacement.

**Conclusions:**

In this case, proximal SINE occurred 6 years after TEVAR. Because SINE may occur even in the long term after TEVAR, careful follow-up is necessary.

## Background

Stent graft-induced new entry (SINE), defined as the stent graft-induced formation of a new entry point for blood to enter an area, is being increasingly observed after thoracic endovascular aortic repair (TEVAR) for Stanford type B aortic dissection worldwide. We herein describe a case of Stanford type A dissection due to proximal SINE after TEVAR for Stanford type B dissection. Careful excision of the entry on the lesser curvature side prevented total arch replacement.

## Case presentation

**Fig. 1:**
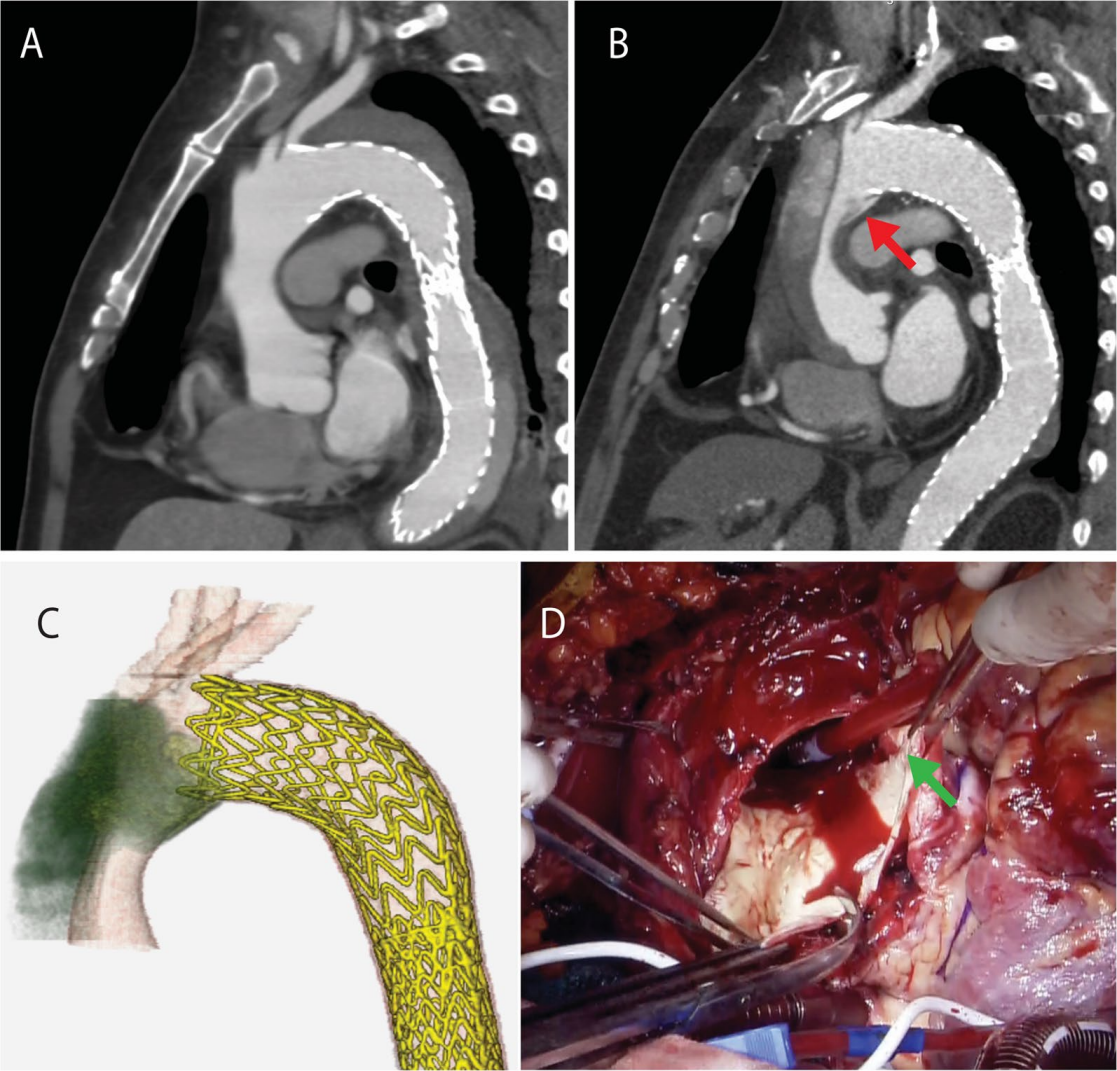
Preoperative and intraoperative images. **A** Computed tomography after thoracic endovascular aortic repair 7 years previously. **B**, **C** Preoperative computed tomography showed proximal stent graft-induced new entry at the aortic arch (red arrow). **D** Intraoperative photograph showed entry (green arrow)

**Fig. 2:**
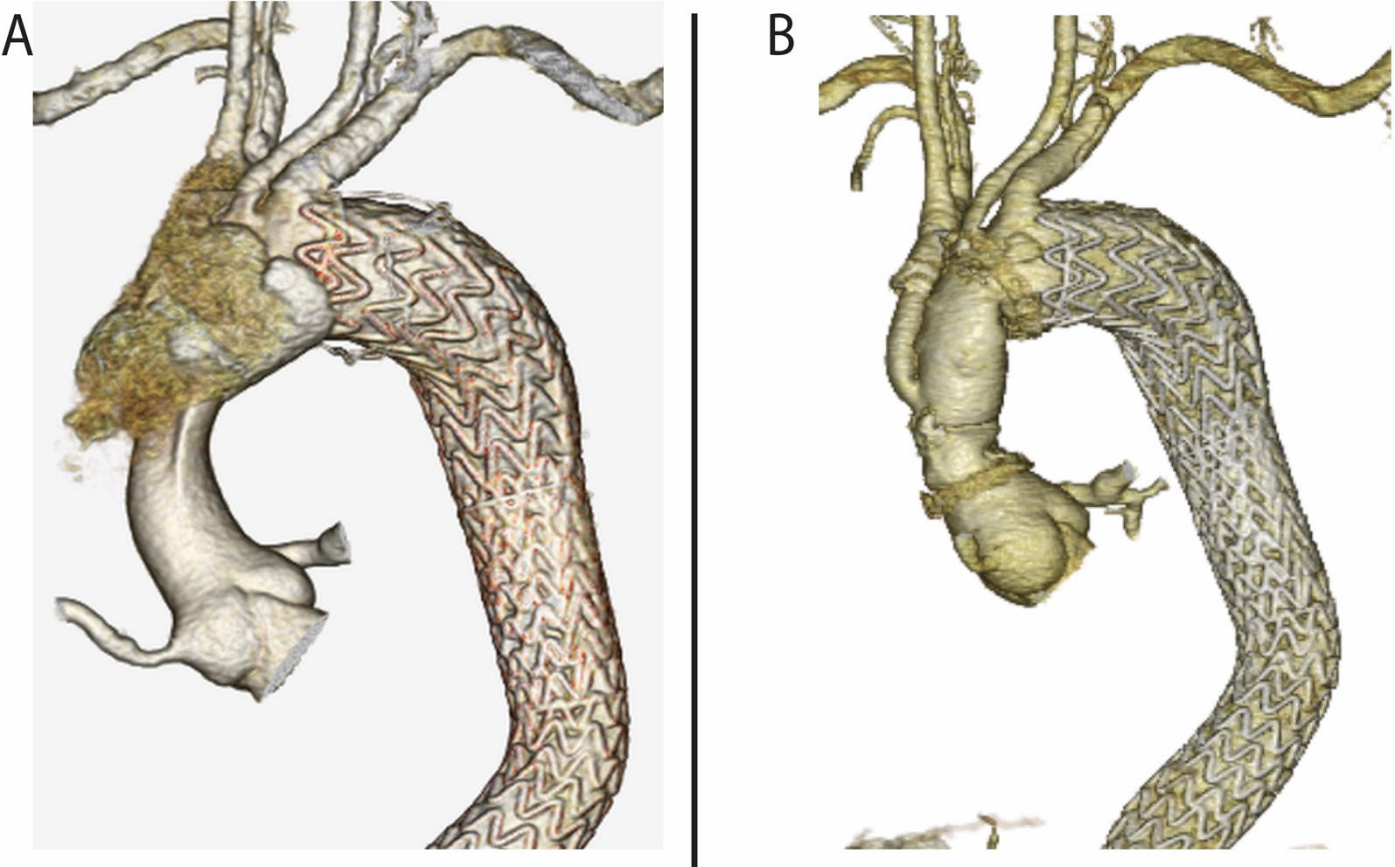
Preoperative and postoperative images. **A** Preoperative computed tomography volume rendering showed type A dissection. **B** Postoperative computed tomography volume rendering showed partial arch graft replacement

A 58-year-old man presented to our hospital because of chest discomfort and a difference in blood pressure between the right and left arms. He had no family history and did not meet the revised Ghent criteria for Marfan syndrome. The patient had undergone TEVAR with a GORE TAG conformable thoracic aortic graft (CTAG) (diameter 40 mm, length 20 cm and diameter 31 mm, length 20 cm) for Stanford type B dissection at 52 years of age. Noninvasive blood pressure measurement showed that the blood pressure in his right arm was 30 mmHg lower than that in his left arm. We suspected Stanford type A dissection. Enhanced computed tomography showed proximal SINE and therefore type A dissection (Figs. 1A–C, 2A). An emergent operation was performed. The patient was placed in the supine position following induction of general anesthesia. After the right femoral artery and vein were exposed, cardiopulmonary bypass was established via cannulation of these vessels. Median sternotomy was then performed. In our hospital, the routine repair technique for aortic dissection is to perform a median sternotomy after starting femoral bypass. The brachiocephalic and left carotid arteries were exposed during systemic cooling. Hypothermic circulatory arrest was performed at a rectal temperature of 28 °C. Cardioplegic arrest was achieved with intermittent administration of retrograde and antegrade cold blood cardioplegia. After insertion of catheters into the innominate and left common carotid arteries, selective antegrade cerebral perfusion was initiated. The entry tear was found at the lesser aortic arch curve, at which the proximal stent was positioned (Fig. 1D). The entry was excluded, and the transverse arch was transected proximal to the left common carotid artery. After reinforcement of the distal end of the aorta with a felt strip, a four-branched, 28-mm Hemashield prosthesis (Getinge, Gothenburg, Sweden) was anastomosed to the distal end using running 4-0 polypropylene sutures. Systemic perfusion was restarted through the side branch of the graft. The total circulatory arrest time was 66 min. Proximal anastomosis was then performed using continuous 4-0 polypropylene sutures with double-felt-strip reinforcement. With the heart beating, reconstruction of the innominate artery was performed using continuous 5-0 polypropylene sutures. The cardiopulmonary bypass and aortic cross-clamp times were 250 and 139 min, respectively. Postoperative computed tomography showed exclusion of the entry (Fig. 2B). Partial rupture of the tunica media elastic fibers and deposition of mucin were identified, indicating connective tissue disease. Although the patient’s postoperative course was uneventful, weight management and drug adjustment for paroxysmal atrial fibrillation took time; thus, he was discharged on postoperative day 27.

## Discussion

SINE is a major complication of TEVAR [1]. New intimal tears after stent graft insertion can be caused by aortic wall fragility or irritation caused by the stent graft [2]. SINE is generally believed to develop in patients with acute or chronic aortic dissection [3]. In the acute phase, because the intimal membrane is thinner and stretches more easily, it can expand and recover its original shape very easily after TEVAR [1]. In the chronic phase, however, the intimal membrane is thicker and more fibrotic than in acute aortic dissection. For this reason, the expansile force of the stent graft persistently injures the rigid true lumen [4]. The intima can then ultimately develop new intimal tears, resulting in aortic dissection.

In this case, SINE had developed in the proximal site 6 years after the first TEVAR. In past reports, SINE had occurred in the acute phase or up to 3 years postoperatively; this is the first report of SINE developing after 6 years postoperatively [5, 6]. Ma et al. [7] reported that a > 15% oversize ratio at the first TEVAR for aortic dissection and a stent graft length of < 145 mm were risk factors for SINE. Our patient did not have these risk factors. Our case is considered rare because Ma et al. [7] reported that a CTAG positioned at Z3 resulted in SINE less frequently than when other TEVAR devices were used.

Hemiarch replacement was considered, and we judged that the margin of the aortic anastomosis in the proximal site from the innominate artery could not be ensured when the entry was removed. We decided to perform partial arch replacement to reconstruct the innominate artery. As a result, we avoided total arch replacement. If we had trimmed the distal anastomosis site at Z3, we might have faced the complicated problem whether we cut the CTAG strad.

## Conclusions

In this case, proximal SINE occurred 6 years after TEVAR. Because SINE may occur even in the long run after TEVAR, careful follow-up is necessary.

## Data Availability

Not applicable.

## Abbreviations

CTAG: Conformable thoracic aortic graft
SINE: Stent graft-induced new entry
TEVAR: Thoracic endovascular aortic repair
